# Supplementary material for: A set of multi-entry identification keys to African frugivorous flies (Diptera, Tephritidae)
Source: Zookeys. 2014 Jul 24;(428):97–108. doi: 10.3897/zookeys.428.7366 (PMC4143993; doi:10.3897/zookeys.428.7366)
Supplement: Supplementary material 6 — Key to Ceratitis [file zookeys-428-097-s006.zip › SF6_ZooKeys_key to Ceratitis/key/SF6_ZooKeys_key to Ceratitis/Media/Html/Ceratitis lineata.htm]

Ceratitis (Ceratalaspis) lineata (Hering)


***Ceratitis*** **(*Ceratalaspis*)
*lineata* (Hering)**

*Pardalaspis lineata* Hering, 1938: 406.

Body
length: 4.60 mm; wing length: 4.50 mm.

Male

(Based
on original description, with additions based on KBIN and USNM specimens and
indicated as such) Head. Antenna yellow to yellow-orange. Third antennal
segment broken in holotype, less than three times as long as second segment in
USNM specimen. Arista with short hairs over entire length. Frons (in material
studied) yellow, with scattered hairs which are distinctly darker than frons,
in lateral view not distinctly projecting forwards at antennal implant; face
yellow-white, lower margin near mouth and below eyes more yellow. Chaetotaxy
normal for subgenus.

Thorax.
Postpronotum yellow-white, spotted. Ground colour of mesonotum pale with orange
tinge. Inner scapular setae missing, outer scapular setae black. One
anepisternal bristle, in USNM specimen on one side second lower bristle which
is less developed. Scutellum white basally, otherwise yellow with three black
separate markings apically; basally two dark separate spots. Subscutellum with
three black spots, widely separated, dorsally connected.

Wing
bands with markings extensively yellow. Banding, marginal band continuous;
cubital band free; medial band absent; crossvein r-m at or just before middle
of discal cell. Crossvein dm-cu posteriorly more inwards than anteriorly.

Legs
yellow; setation typical for subgenus, mainly pale especially on femora.
Posterior and posterodorsal rows on fore femur pale. Ventral spines dark in USNM
specimen.

Abdomen
yellow. Setation and banding typical for subgenus.

Female

Unknown.

(Description
after De Meyer, 1998)
